# COL1A1 is a prognostic biomarker and correlated with immune infiltrates in lung cancer

**DOI:** 10.7717/peerj.11145

**Published:** 2021-03-30

**Authors:** Qishun Geng, Zhibo Shen, Lifeng Li, Jie Zhao

**Affiliations:** 1 Department of Pharmacy, The First Affiliated Hospital of Zhengzhou University, Zhengzhou, China; 2 Engineering Laboratory for Digital Telemedicine Service, The First Affiliated Hospital of Zhengzhou University, Zhengzhou, China

**Keywords:** Lung cancer, Biomarker, COL1A1, Immunity, Survival

## Abstract

**Objective:**

Lung cancer (LC) is one of the top ten malignant tumors and the first leading cause of cancer-related death among both men and women worldwide. It is imperative to identify immune-related biomarkers for early LC diagnosis and treatment.

**Methods:**

Three Gene Expression Omnibus (GEO) datasets were selected to acquire the differentially expressed genes(DEGs) between LC and normal lung samples through GEO2R tools of NCBI. To identify hub genes, the DEGs were performed functional enrichment analysis, the protein–protein interaction (PPI) network construction, and Lasso regression. Then, a nomogram was constructed to predict the prognosis of patients with carcinoma based on hub genes. We further evaluated the influence of COL1A1 on clinical prognosis using GSE3141, GSE31210, and TCGA database. Also, the correlations between COL1A1 and cancer immune infiltrates and the B7-CD28 family was investigated via TIMER and GEPIA. Further analysis of immunohistochemistry shown that the COL1A1 expression level is positively correlated with CD276 expression level.

**Results:**

By difference analysis, there were 340 DEGs between LC and normal lung samples. Then, we picked out seven hub genes, which were identified as components of the risk signature to divide LC into low and high-risk groups. Among them, the expression of COL1A1 is highly correlated with overall survival(OS) and progression-free survival (PFS) (*p* < 0.05). Importantly, there is a moderate to strong positive relationships between COL1A1 expression level and infiltration level of CD4+ T cells, Macrophage, Neutrophil, and Dendritic cell, as well as CD276 expression level.

**Conclusion:**

These findings suggest that COL1A1 is correlated with prognosis and immune infiltrating levels, including CD4+ T cells, Macrophage, Neutrophil, and Dendritic cell, as well as CD276 expression level, indicating COL1A1 can be a potential immunity-related biomarker and therapeutic target in LC.

## Introduction

Lung cancer (LC) is one of the top ten malignant tumors and the first leading cause of cancer-related death worldwide, which is a major global health problem (Bray et al., 2018; Ferlay et al., 2019; Sung, Ferlay & Siegel, 2021). In recent years, due to air pollution, smoking and other factors, the incidence and mortality of lung cancer have increased rapidly, making it one of the most dangerous tumors to human health. Lung cancer is also the most important type of occupational cancer (Rocco et al., 2018). Studies have shown that there is a certain relationship between high-risk occupational exposure and the occurrence of LC, and such patients are more likely to have extra-pulmonary lesions, accompanied by respiratory diseases, which have more difficult to treat (Wong et al., 2019). At present, the treatment of advanced lung cancer mainly includes chemotherapy, radiotherapy, targeted therapy and immunotherapy. The advent of targeted therapy and immunotherapy has brought the treatment into the era of ‘individualized treatment’ and ‘precision treatment’. Despite great advances in diagnostic and therapeutic techniques, due to the high recurrence and metastasis, median overall survival (OS) and the 5-year survival rate of patients with LC still very low (Ferlay et al., 2019). Therefore, it is significant for enhancing the prognosis of lung tumor patients to detect prognostic marker and potential drug targets.

Suppressing the immune system checkpoint, activating T cells and B cells to kill tumor cells, provides a new idea for tumor immunotherapy. During the decade, tumor-specific immunotherapy has made significant progress, of which the research progress of the B7-CD28 family is particularly prominent (Tanaka & Sakaguchi, 2017). The B7-CD28 costimulatory pathway is one of the most important secondary signal transduction mechanisms. It is essential for maintaining a delicate balance between immunity and suppression of autoimmunity, and has great potential in tumor therapy (Schildberg et al., 2016; Sharpe & Freeman, 2002). Although, immunotherapy has made great contributions to improving the prognosis of LC patients, and tumor-specific immunotherapy has been widely used in the clinic, there are still some problems with this treatment, including adverse reactions and drug resistance (Fesnak, June & Levine, 2016). To reduce the adverse reactions of immunotherapy and overcome rapid drug resistance, experts in the field of oncology at home and abroad have made unremitting efforts. Through basic research and clinical verification, most of the current experts believe that combined immunotherapy is the main trend of cancer treatment in the future. Previous studies have shown that immune combined targeted therapy can significantly improve the overall survival (OS) and progression-free survival (PFS) of LC patients (Hellmann et al., 2018). Hence, immune combined targeted therapy should be investigated to enhance prognosis and individualized treatments.

In this study, Collagen type I alpha 1 chain (COL1A1) was detected as the prognostic marker and potential drug target in lung tumor by screening different expression genes (DEGs) based on GEO datasets. Based on GSE31210, GSE3141 and TGA databases, it is shown that COL1A1 is closely related to the prognosis of lung cancer patients. Moreover, we investigated the correlation of COL1A1 with the B7-CD28 family in the lung tumor (Fig. S1 ). The findings suggest COL1A1 played a crucial part in the prognostic of lung tumor as well as reveal the potential mechanism between COL1A1 and tumor-immune, which lay the foundation for clinical research and immune targeted therapy.

## Materials and Methods

### Data source

DEGs transcriptome data from lung tumor in TCGA dataset were obtained from the website of the Cancer Genomics Browser of the University of California Santa Cruz, including lung tumor samples(*n* = 1019) and normal lung samples(*n* = 110). Besides, NCBI-GEO, a free public database of transcriptional expression profile, stores original submitter-supplied records (Series, Samples and Platforms) as well as curated Datasets. We obtained the gene expression profile of GSE32867 (Selamat et al., 2012), GSE43458 (Kabbout et al., 2013) and GSE116959 (MorenoLeon et al., 2019) in lung cancer and normal lung tissues. The data of GSE32867 were obtained with the GPL6884 platform and came from 58 lung tumours and 58 normal lung tissue sample. Similarly, the data of GSE43458 were based on the GPL6244 platform. The gene microarray data were collected from 80 lung adenocarcinoma and 30 non-tumors. The GSE116959 data were obtained from the GPL17077 platform, with 57 primary lung tumors and 11 adjacent Normal lung tissues.

### The acquisition of DEGs (different expression genes)

We got the table of genes ordered by significance between lung carcinoma and normal lung samples of each gene expression profiles through the GEO2R online analysis tool of NCBI (https://www.ncbi.nlm.nih.gov/geo/geo2r/). According to the cutoff standard requirements (adjusted *P* < 0.05 and —logFC— ≥1.0) , we got the DEGs between lung carcinoma and normal lung samples. Then, in order to obtain the common DEGs of three gene expression profiles we employed Venn software online to plot the Venn diagram of the DEGs.

### PPI (protein–protein interaction) network and module analysis

DEGs-encoded proteins and the PPI were obtained using the online database STRING (http://string-db.org) (Szklarczyk et al., 2019). Then, Cytoscape (version 3.7.1) (Smoot et al., 2011), a public source bioinformatics software platform, was used to visualize and analyze molecular interaction networks. Cytoscape’s plug-in Molecular Complex Detection(MCODE) (version 1.5.1) is an APP for clustering a given network based on topology to find tightly connected regions. Then, by means of calculating the degree of connectivity between DEGs, the hub genes in the PPI networks were identified using a plugin, namely CytoHubba (Chin et al., 2014), in Cytoscape software (Version3.6.1). The PPI network was drawn using Cytoscape, and the most important module in the PPI network was identified by MCODE with criteria as follows: degree cut-off = 2, node score cut-off = 0.2, Max depth = 100, and K-score = 2. In addition, ClueGO (version 3.0.3) (Bindea et al., 2009) is a plug-in of Cytoscape for visualizing the nonredundant biological terms for large clusters of genes in a functionally grouped network, which was used to perform the biologic process functional annotation analysis of hub genes.

### The correlation and nomograms of key member in DEGs

According to TCGA data, we compared the co-expression of DEGs in normal tissues and tumor tissues, and further compared the correlation of DEGs in lung tumor sample. Based on optimal *λ* value, we performed the Lasso regression to construct a predictive module and ensure that the multifactor model was not overfitting. Then, the receiver operating characteristic curves (ROC) were utilized to assess the discrimination and accuracy of the model.

### Survival analysis

The Kaplan–Meier plotter database (http://kmplot.com), an online database capable of assessing the 54,675 genes on survival in 21 cancer types, was used to evaluate the prognostic roles of significant DEGs identified in this study. The database simultaneously integrates gene expression and clinical data downloaded from GEO and TCGA, which calculate the hazard ratio with 95% CIs and log-rank *p*-value by a Kaplan–Meier survival plot. Log-rank *p*-value <0.05 was considered as statistically significant. What’s more, the expression, survival and ROC analysis of COL1A1 was assessed in lung carcinoma cohort from the TCGA database. In addition, multiple databases including the Gene Expression Omnibus (GEO) (ID: GSE3141, ID: GSE31210) were used for survival analysis to validate the survival value of COL1A1.

### Immunity correlation analysis

TIMER (Li et al., 2017), a comprehensive resource for systematical analysis of immune infiltrates across diverse cancer types, was used to assess the correlations of COL1A1 expression with immune infiltration levels in LUAD and LUSC. The coexpression analysis of COL1A1 and B7 family member (including CD274, CD80, CD86, CD276, CD273, CD275, B7-H4, B7-H5, CD28, B7-H7, CD152, CD279, CD278, TLT-2 and NKp30) was assessed in normal lung cohort and lung carcinoma cohort from the TCGA database. Then, the correlation between COL1A1, B7-H4 and CD276 was further analyzed in lung carcinoma cohort. The Gene Expression Profiling Interactive Analysis (GEPIA; http://gepia.cancer-pku.cn/detail.php) (Tang et al., 2017), a newly developed database that integrated The Cancer Genome Atlas (TCGA) and the Genotype-Tissue Expression project, can provide reliable correlation between genes. TIMER was applied to plot the expression scatterplots between a pair of genes in a given cancer type, together with the Spearman correlation and estimated statistical significance.

### Immunohistochemistry (IHC) Staining

To examine COL1A1 and CD276 expression in tumors and matched normal tissue, samples from cancer patients were obtained from lung cancer arrays (HLugA030PG02, SHANGHAI OUTDO BIOTECH Co., Ltd. Shanghai, China). Tumor tissues chip were deparaffinized and rehydrated, followed by antigen retrieval. The sections were then blocked with 5% BSA in PBS and incubated with COL1A1 antibody (1 mg/mL, 1:200; Affinity) and CD276 antibody (1 mg/mL, 1:200; Affinity) at 4 °C overnight. After washing with 0.01 mol/L PBS for 5 min ×3 times, the operation was performed according to the instructions of the ultra-sensitive SP immunohistochemistry kit. 3, 3-diaminobenzidine tetrahydrochloride (DAB) was used for color development and hematoxylin was used to restain the nucleus. Dyeing intensities are classified as follows: 0, negative; 1, weak; 2, moderate; or 3, strong.

### Statistical analysis

A part of statistical analyses was performed by default as described by web resources. Other statistical analyses were performed with GraphPad Prism 8 and R version 3.6.1 software (Institute for Statistics and Mathematics, Vienna, Austria; https://www.r-project.org) (Package: ggplot2, rms, glmnet, pheatmap, survival, ROCR etc.). Only *P* value< 0.05, adjusted *P* < 0.05 and Log-rank *p* value<0.05 were thought to be statistical significance.

## Results

### Identification of DEGs in lung cancer

**Figure 1:**
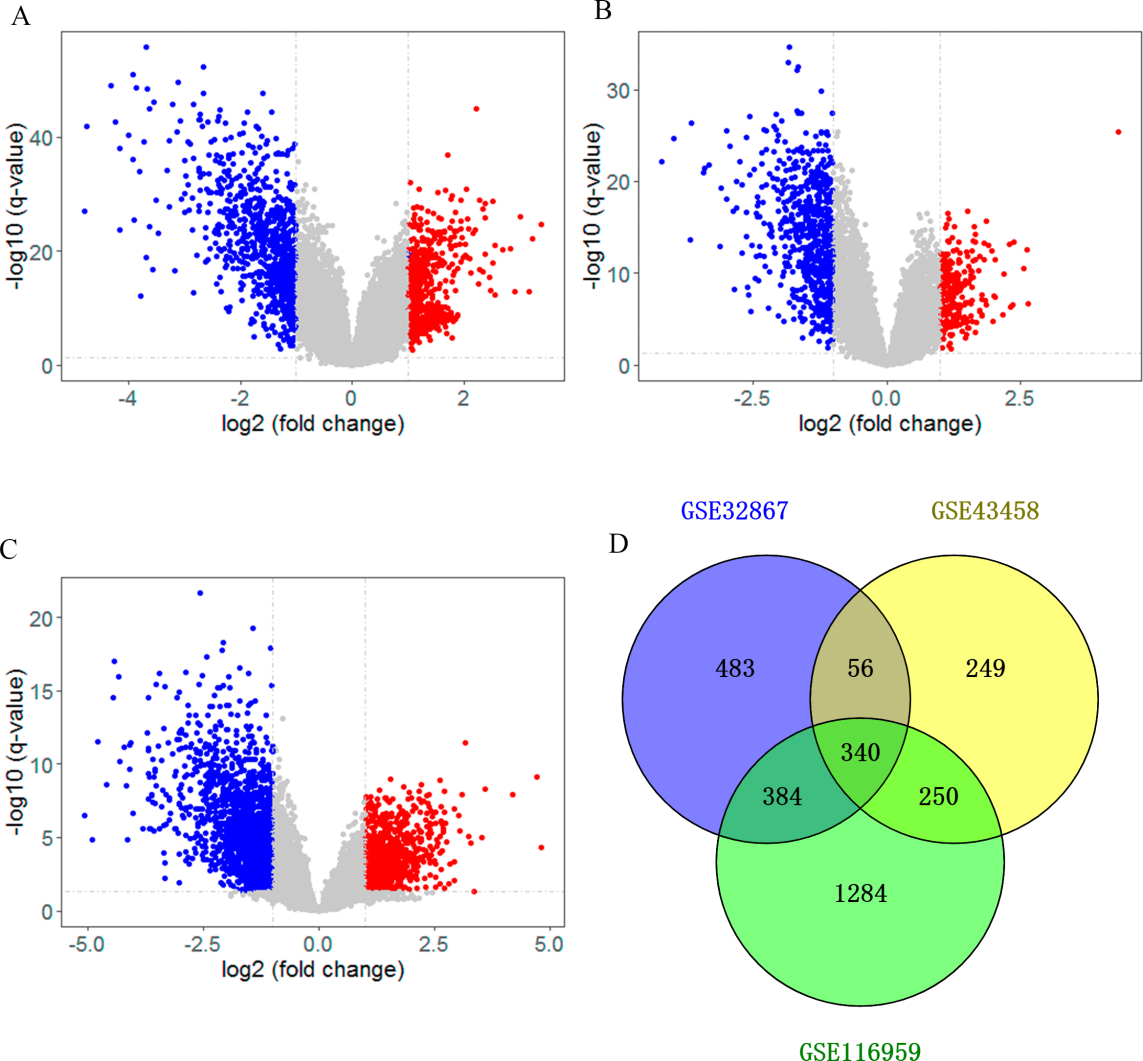
Identification of differentially expressed genes (DEG). Volcano plot showing the differentially expressed genes of (A) GSE32867, (B) GSE43458 and (C) GSE116959. (D) Veen diagram of common DEGs to three gene expression profiles.

After searching the appropriate datasets in GEO database according to the eligibility criteria, three genome-wide gene expression datasets (GSE32867, GSE43458 and GSE116959) regarding lung cancer were chosen as discovery set. As shown in the volcano plot, DEGs in those datasets were identified separately with a threshold of adjust *p* < 0.05 and —log2FC— >1 (Figs. 1A–1C). In GSE32867 dataset, a total of 1263 DEGs were screened out. In the GSE43458 dataset, there were 895 DEGs in lung cancer tissues compared with normal control samples. For GSE116959 dataset, there were 2258 DEGs were identified. After the DEGs in three datasets were intersected, we totally identified 340 DEGs that were commonly appeared in three datasets, including 268 downregulated genes (log2FC < -1) and 72 up-regulated genes (log2FC > 1) in the lung tissues (Fig. 1D, Table S1), which were selected for subsequent analyses.

### Construction and analysis of PPI network

**Figure 2:**
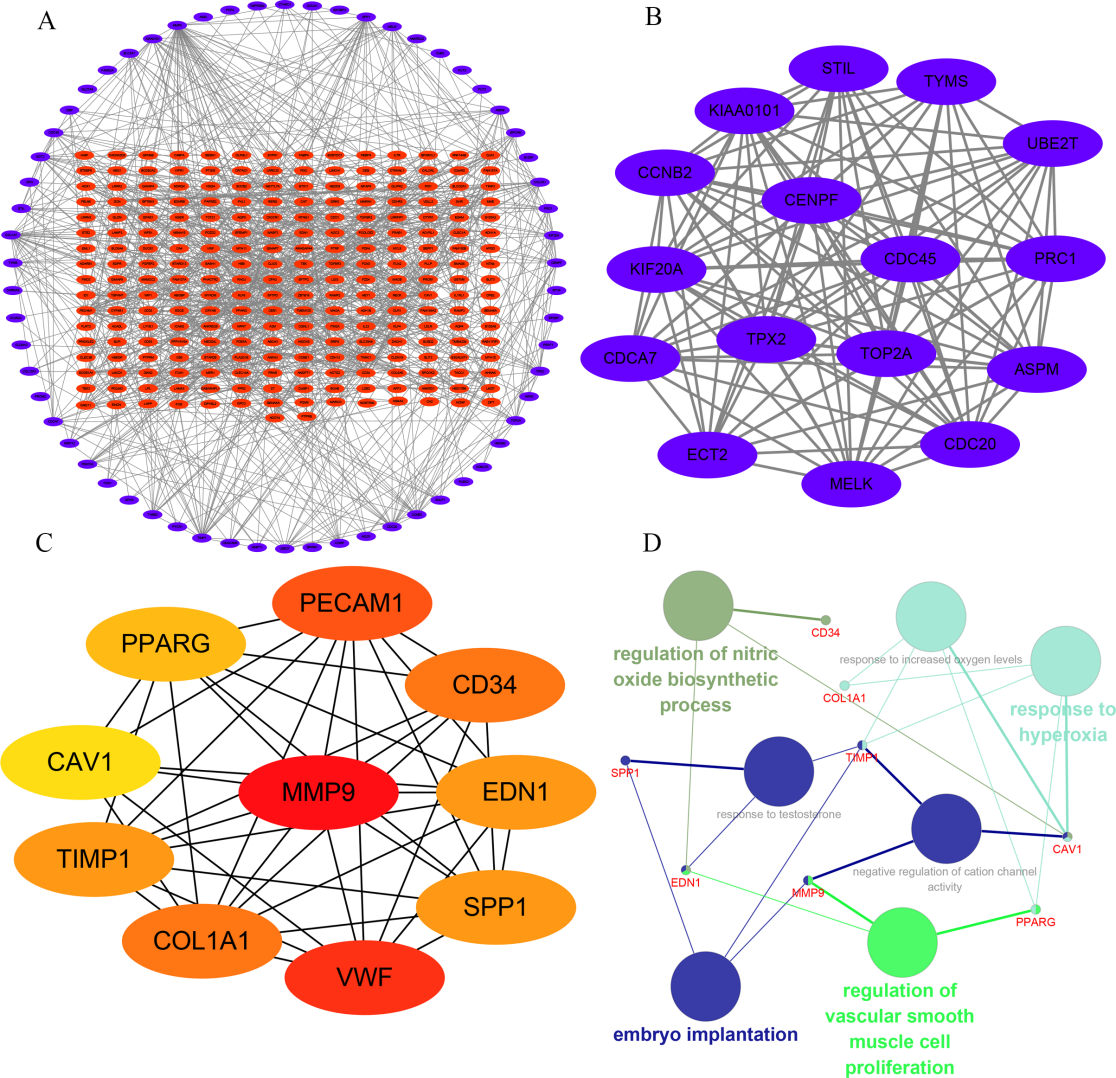
Interaction network and analysis of the hub genes. (A) PPI network constructed with the DEGs. The blue points represent upregulated genes screened on the basis of fold change > 1.0 and a corrected *P*-value of < 0.05. The red points represent downregulation of the expression of genes screened on the basis of fold change <1.0 and a corrected *P*-value of < 0.05. (B) The most significant module was obtained from PPI network with 16 nodes and 114 edges. (C) The hub genes were identified using Cytoscape. (D) The biological process analysis of hub genes was performed using the BiNGO plug-in, ∗*p* < 0.05. Different colors of nodes refer to the functional annotation of ontologies.

In order to understand the mutual interaction of the identified DEGs, the STRING database and Cytoscape software were used to constructed a PPI network, which shown the complicated interactions among multiple genes (Fig. 2A). The most important module was obtained using Cytoscape (MCODE plug-in) (Fig. 2B), which was performed functional enrichment analysis by the Database for Annotation, Visualization and Integrated Discovery (DAVID). The result indicated the module participated in nucleotide binding, cell cycle and cell division (Table 1), which are closely related to tumor occurrence and progression. This further highlights the importance of the 340 common DEGs in tumor treatment. Then, the top ten genes, including SPP1, TIMP1, MMP9, COL1A1, PPARG, EDN1, CAV1, PECAM1, VWF and CD34, were identified as hub genes according to the degree score generated by CytoHubba plug-in (Fig. 2C, Table 2). The biological process analyses of hub genes were analyzed using BINGO plug-in, showing hub genes participate in regulation of nitric oxide biosynthetic process, regulation of vascular smooth muscle cell proliferation, embryo implantation, response to hyperoxia and response to testosterone (Fig. 2D). These results indicate that the 10 hub genes play a vital role in the regulation of biological processes and related pathways, and may become an important target for the treatment of LC.

**Table 1:** Significantly enriched GO terms of the common DEGs (BP, biological process; CC, cellular component; DEG, differentially expressed gene; GO, Gene Ontology; MF, molecular function).

**Gene symbol**	**Full name**	**Function**
MMP9	Matrix Metallopeptidase 9	Pathways: IL-17 Family Signaling Pathways and TNF signaling pathway; GO: endopeptidase activity and hydrolase activity.
VWF	Von Willebrand Factor	Pathways: RET signaling and Complement and coagulation cascades; GO: immunoglobulin binding and collagen binding.
PECAM1	Platelet And Endothelial Cell Adhesion Molecule 1	Pathways: NF-kappaB Signaling and Innate Immune System; GO:protein binding and protein homodimerization activity
CD34	CD34 Molecule	Pathways: NF-kappaB Signaling and Cell adhesion molecules (CAMs);GO: transcription factor binding and carbohydrate binding.
COL1A1	Collagen Type I Alpha 1 Chain	Pathways: VEGF Signaling Pathway and ERK Signaling; GO: extracellular matrix structural constituent and extracellular matrix structural constituent conferring tensile strength.
SPP1	Secreted Phosphoprotein 1	Pathways: ERK Signaling and TGF-beta Receptor Signaling; GO: cytokine activity and integrin binding.
TIMP1	TIMP Metallopeptidase Inhibitor 1	Pathways: GPCR Pathway and CREB Pathway; GO: enzyme inhibitor activity and peptidase inhibitor activity
EDN1	Endothelin 1	Pathways: RET signaling and TNF signaling pathway; GO: cytokine activity and signaling receptor binding.
PPARG	Peroxisome Proliferator Activated Receptor Gamma	Pathways: AMP-activated Protein Kinase (AMPK) Signaling and PPAR signaling pathway; GO: activating transcription factor binding and DNA-binding transcription factor activity.
CAV1	Caveolin 1	Pathways: RET signaling and ALK1 signaling events ; GO: peptidase activator activity and patched binding.

**Table 2:** Hub genes with higher degree of connectivity.

**Term**	**Description**	**Count in gene set**	*P*-Value
**Molecular function**			
GO:0000166	nucleotide binding	6	1.05E−02
GO:0017076	purine nucleotide binding	5	3.22E−02
GO:0032553	ribonucleotide binding	5	2.78E−02
GO:0032555	purine ribonucleotide binding	5	2.78E−02
GO:0001882	nucleoside binding	5	1.78E−02
GO:0001883	purine nucleoside binding	5	1.74E−02
GO:0030554	adenyl nucleotide binding	5	1.65E−02
GO:0032559	adenyl ribonucleotide binding	5	1.38E−02
GO:0005524	ATP binding	5	1.31E−02
**Cellular Component**			
GO:0043228	non-membrane-bounded organelle	9	6.44E−04
GO:0043232	intracellular non-membrane-bounded organelle	9	6.44E−04
GO:0005856	cytoskeleton	9	6.05E−06
GO:0015630	microtubule cytoskeleton	9	4.68E−09
GO:0044430	cytoskeletal part	8	7.06E−06
GO:0031974	membrane-enclosed lumen	6	2.09E−02
GO:0043233	organelle lumen	6	1.93E−02
GO:0070013	intracellular organelle lumen	6	1.76E−02
GO:0031981	nuclear lumen	6	7.44E−03
GO:0005829	cytosol	5	2.92E−02
GO:0005819	spindle	5	7.74E−06
**Biological Process**			
GO:0007049	cell cycle	7	7.05E−05
GO:0022402	cell cycle process	6	1.83E−04
GO:0022403	cell cycle phase	6	4.17E−05
GO:0000278	mitotic cell cycle	6	2.43E−05
GO:0000279	M phase	6	1.38E−05
GO:0051301	cell division	5	1.87E−04
GO:0048285	organelle fission	5	7.01E−05
GO:0000087	M phase of mitotic cell cycle	5	6.43E−05
GO:0000280	nuclear division	5	5.99E−05
GO:0007067	mitosis	5	5.99E−05

### The correlation and nomograms of key member in DEGs

**Figure 3:**
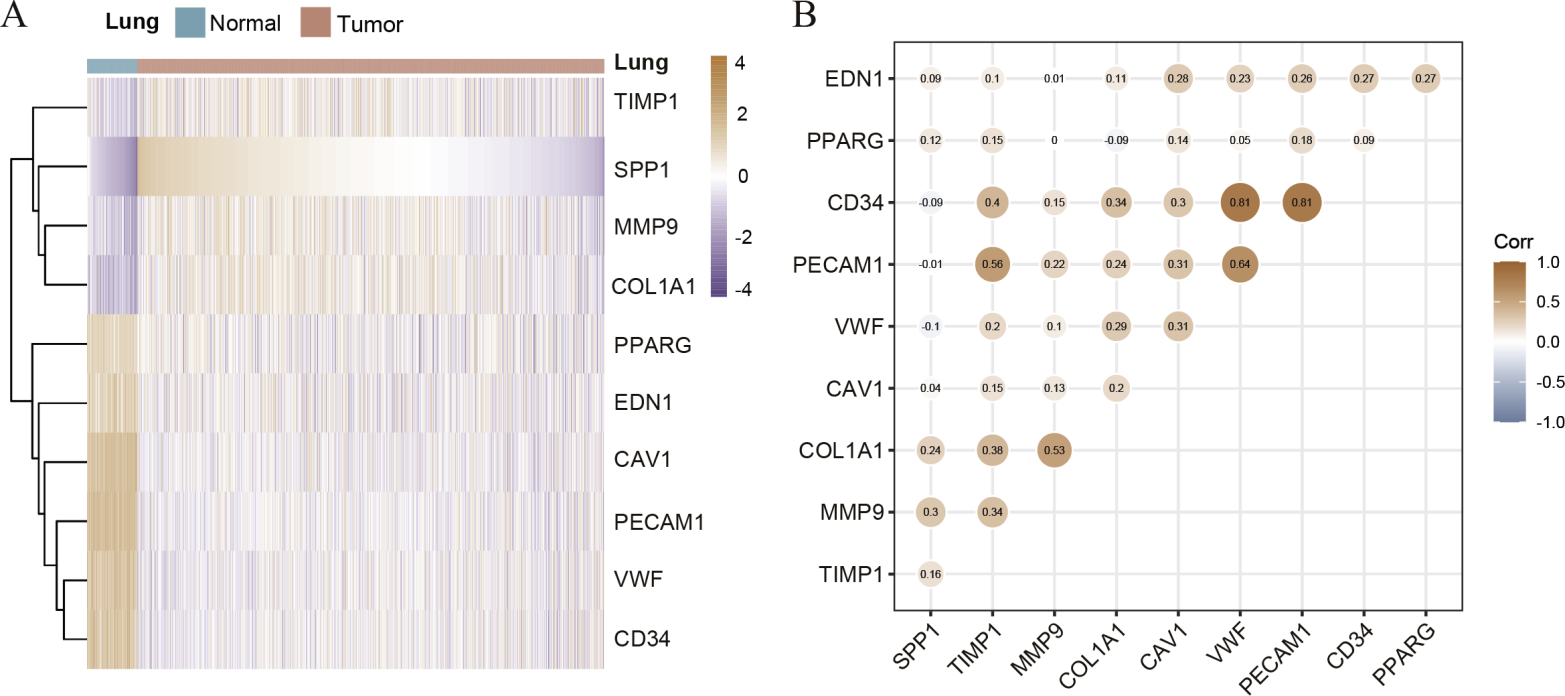
The correlation of hub genes. (A) The hierarchical clustering of hub genes was constructed using TCGA database. (B) The correlation of hub genes in LC cohort.

Hierarchical clustering of the hub genes was performed based on TCGA database (Fig. 3A), indicating the expression pattern across 10 genes, which are consistent with the results of GEO database difference analysis. Compared with normal tissues, the expression of SPP1, TIMP1, MMP9, and COL1A1 is up-regulated in LC tissues; the expression of PPARG, EDN1, CAV1, PECAM1, VWF and CD34 is down-regulated. Furthermore, we analyzed the correlation of the expression of hub gene in lung cancer tissues, of which PECAM1, VWF, CD34, COL1A1, MMP9 and TIMP1 are closely related (Fig. 3B). In order to investigate the prognostic value of hub genes, the Lasso regression was used to screen potential hub genes, indicating that SPP1, TIMP1, COL1A1, PPARG, EDN1, CAV1 and PECAM1 were essential for modeling (Figs. S2A–S2B). In addition, the ROC were applied to evaluate the accuracy and discrimination of modeling, showing low accuracy (Area Under Curve (AUC) of 1-year survival: 0.603; AUC of 5-year survival: 0.577) (Fig. S2C), indicating some potential hub genes expression may not be closely related to the survival of lung cancer patients. Then, the Risk factor association diagram represents the difference in population proportion, survival time (alive/death), and gene expression distribution between high-risk and low-risk groups distinguished by the cox risk model (Fig. S2D).

### Survival analysis of potential hub genes

**Figure 4:**
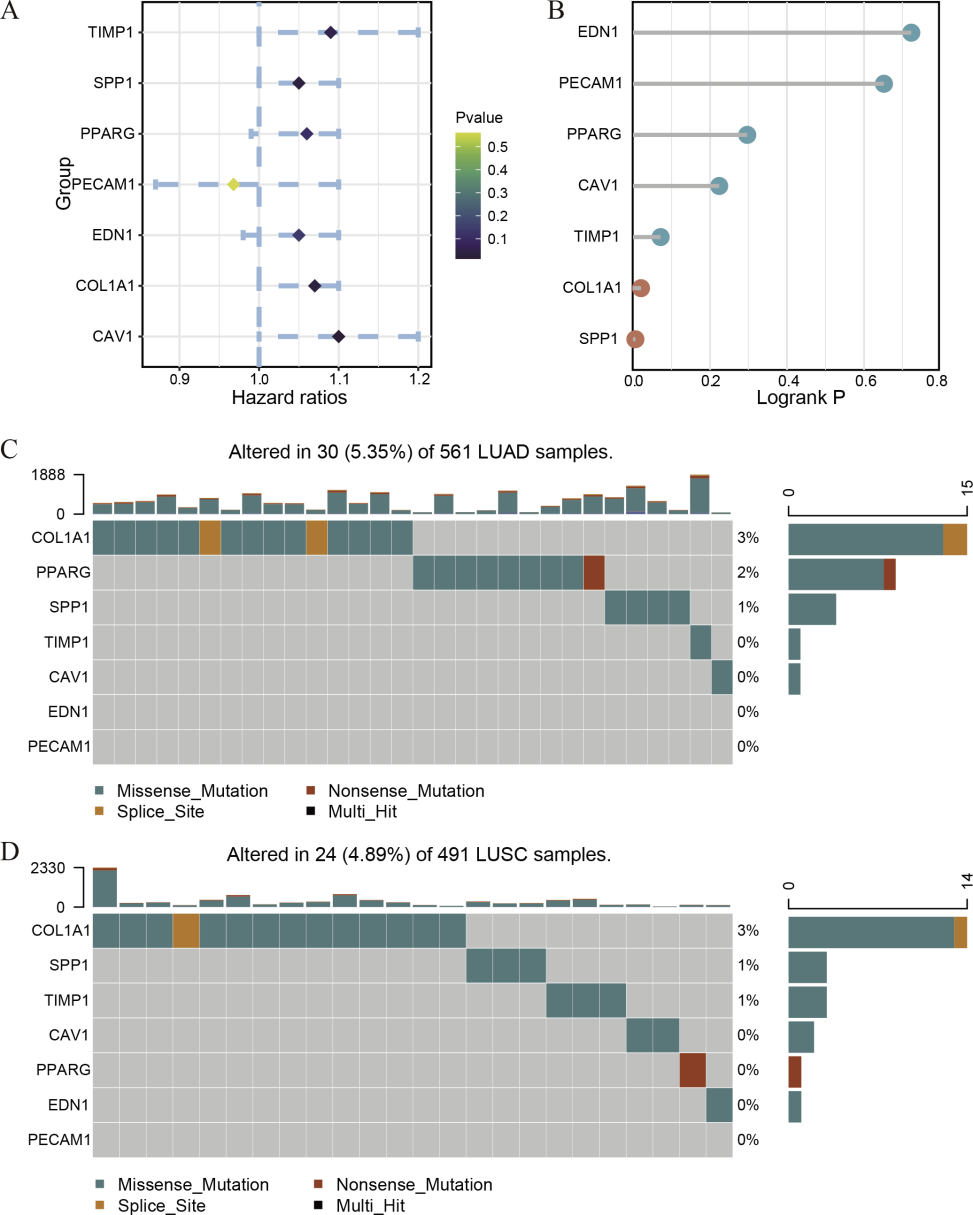
The survival correlation and mutation status of hub genes. The Univariate Cox regression (A) and Logrank regression (B) of hub genes based on TCGA lung tumor dataset. The mutation status of hub genes in LUAD (C) and LUSC (D) based on the TCGA dataset.

**Figure 5:**
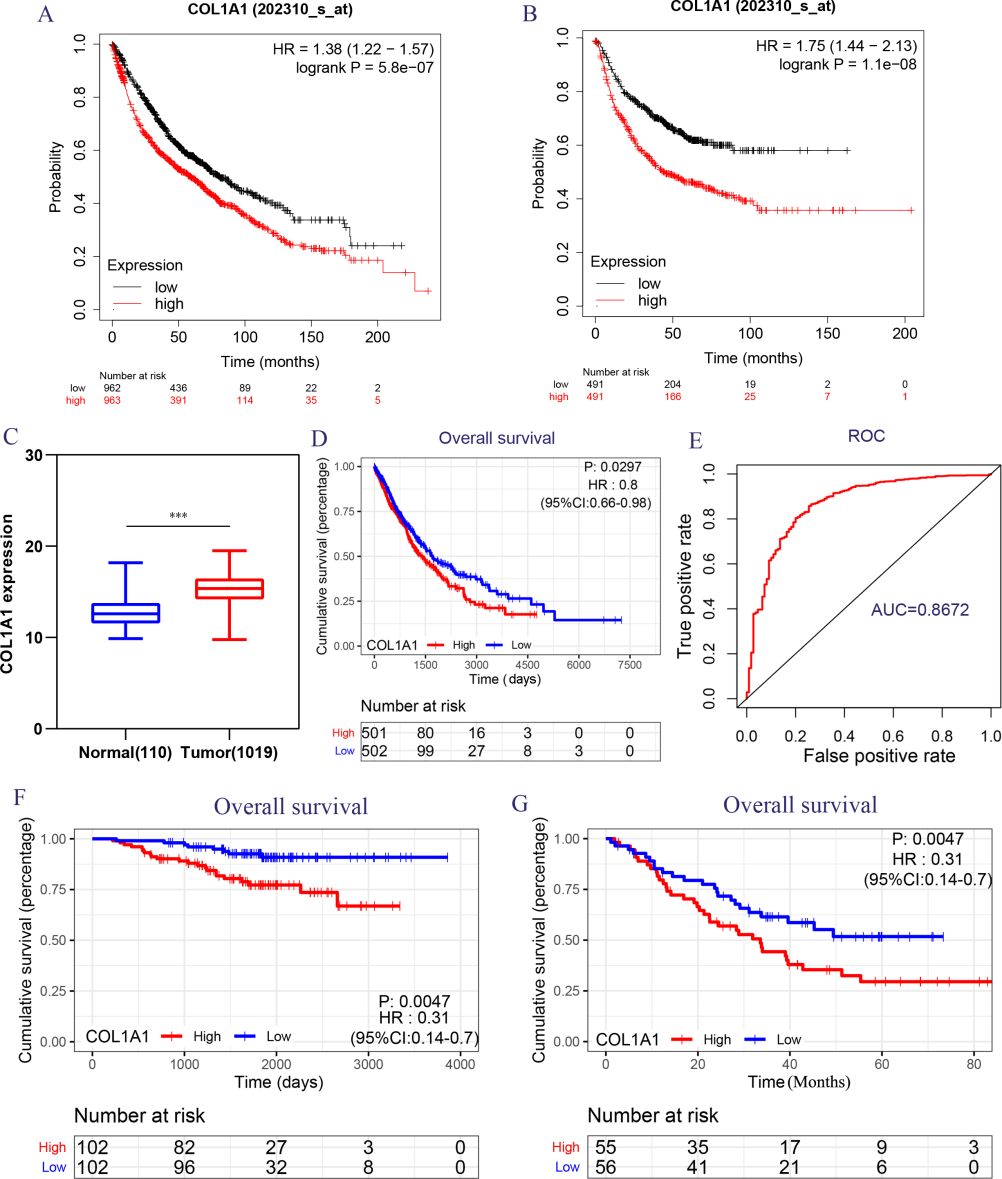
Transcriptional expressions of COL1A1 significantly correlated with poor survival outcomes in LC patients from the TCGA cohort. The high COL1A1 expression correlated with high HR for OS (A) and PFS (B) of LC. (C–D) The expressions of COL1A1, the correlation between the expression of COL1A1 and OS in LC patients from the TCGA cohort, ∗*p* < 0.05. (E) ROC curve with an AUC of 0.8672. The correlation between the expression of COL1A1 and OS in GSE31210 (F) and GSE3141 (G) cohorts, ^∗∗^*p* < 0.01.

Because of the poor predictive value of model, we performed correlation analysis with the clinical correlative of lung cancer outcomes in GEO, EGA, and TCGA lung cancer data sets to determine whether the potential hub genes in lung tumor have clinical relevance. The hub genes were performed Univariate Cox regression and Logrank regression based on TCGA lung tumor dataset, indicating COL1A1 and SPP1 is of great significance to prognosis in both regression analyses (Figs. 4A–4B). Besides, the mutation status of hub genes was assessed in LUAD and LUSC based on TCGA datasets, of which COL1A1 mutation rate is higher (Figs. 4C–4D). Therefore, COL1A1 may be the attractive target in the treatment of carcinoma, which participated in the metastasis and was a reliable biomarker and putative therapeutic target in many cancers (Liu et al., 2018; Ma, Chang & Bamodu, 2019; Oleksiewicz et al., 2017). Besides, Kaplan–Meier Plotter also showed a high COL1A1 expression closely correlated with the over-all survival and the progression-free survival of lung cancer patients (Figs. 5A–5B). In order to verify the diagnostic and prognostic value of COL1A1, COL1A1 expression in lung tumors and matched normal tissues from TCGA dataset was analyze which indicated that COL1A1 expression is significantly higher expression in lung tumor tissues than in matched normal samples (Fig. 5C). To investigate the potential significance of COL1A1 expression, we divided the TCGA lung cancer cohort (*n* = 1003) into COL1A1-high and low expression groups that were performed to evaluate the prognostic value. Patients with high COL1A1 expression had an obviously shorter survival time than those patients with low expression (Fig. 5D). Similar results were obtained with the GEO dataset, including of GSE31210 and GSE3141 datasets (Figs. 5F–5G, all *P* < 0.01). In addition, the area under the curve (AUC) of COL1A1 from TCGA data sets was 0.8672 (Fig. 5E), supporting COL1A1 as a diagnostic and prognostic marker in lung carcinoma.

### The correlation between COL1A1 and the inflammatory activities

**Figure 6:**
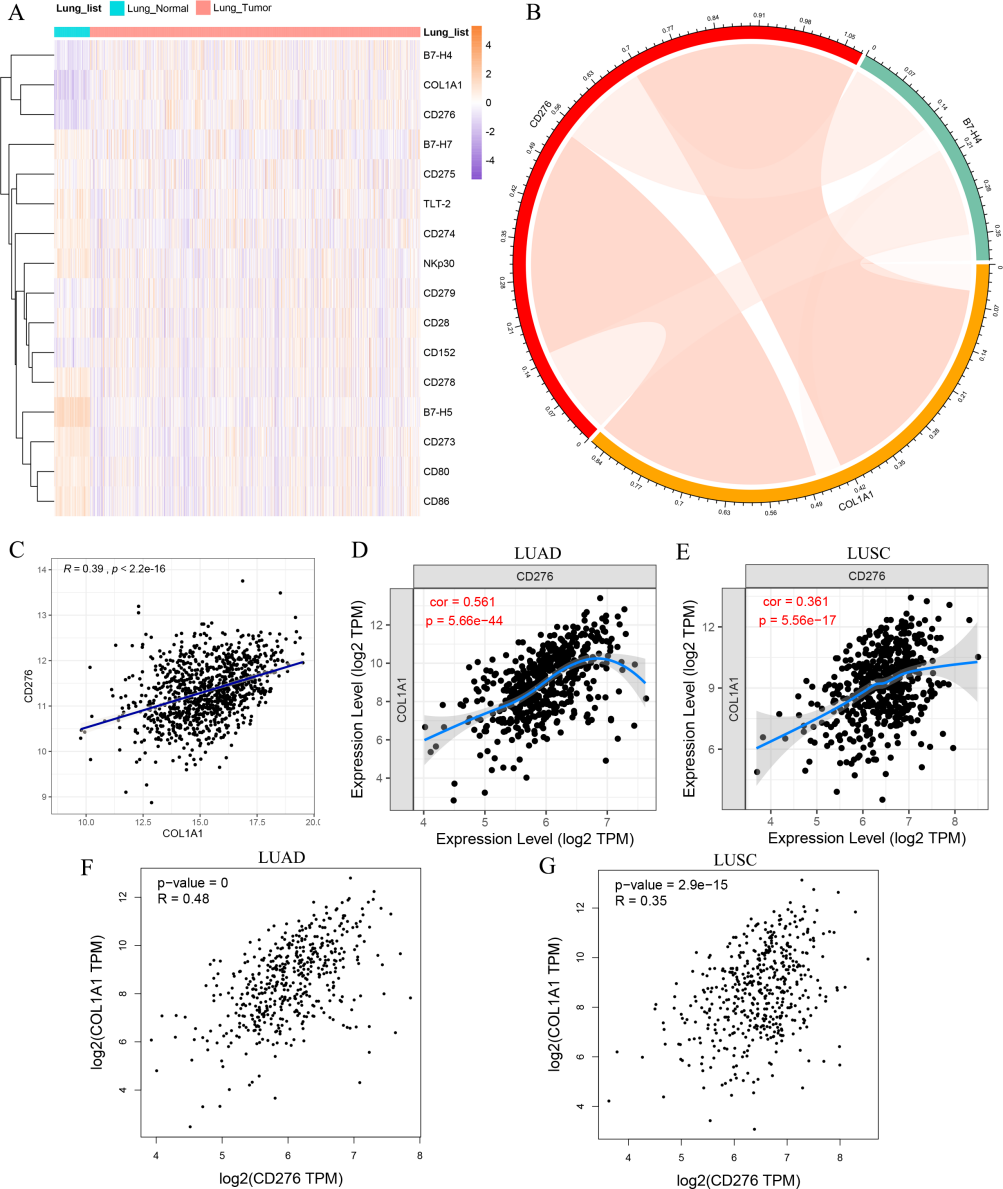
The correlation between COL1A1 and inflammatory activities. (A) The coexpression between COL1A1 and the B7-CD28 ligand–receptor family. (B) The correlation between FN1, CD276 and B7-H4 in the LC cohort. The correlation between COL1A1 and CD276 analyzed by THCA datasets (C), TIMER (D-E) and GEPIA (F–G).

In view of the strongly association between COL1A1 expression and prognosis in lung tumor, we theorized that COL1A1 expression may be regulated by inflammatory responses, contributing to affect the survival rates of tumor patients. The human body needs to control the immune response to obtain the best protective immunity and tolerance. Therefore, TIMER was used to assessed the correlations of COL1A1 expression with immune infiltration levels in LUAD and LUSC. The results indicate that COL1A1 expression has correlations with tumor purity and significant correlations with CD4+ T cells, Macrophage, Neutrophil and Dendritic cell in LUAD and LUSC (Fig. S3). Besides, costimulatory or co-suppressive signals mediated by B7 family molecules and corresponding receptors are involved in the immune regulation of various malignant tumors, and affect tumor progression and metastasis, which is closely related to the malignant degree and prognosis of the disease. Various immunotherapies targeting monoclonal antibodies against B7 family molecules have become the focus of current research (Schildberg et al., 2016). To further study the relationship between COL1A1 and immunity, the coexpression between COL1A1 and the B7-CD28 ligand–receptor family was analyzed in lung cancer and normal lung tissues, including CD274, CD80, CD86, CD276, CD273, CD275, B7-H4, B7-H5, CD28, B7-H7, CD152, CD279, CD278, TLT-2 and NKp30 (Fig. 6A), based on TCGA dataset. The result demonstrated that COL1A1 exhibited a significant co-expression trend with CD276 and B7-H4. Recent studies have shown that CD276 and B7-H4 have a co-inhibitory role on T-cells, participating in tumor cell immune evasion, whose overexpression has a significant relationship with poor prognosis in tumor patients (Altan et al., 2017), which was consistent with the prognostic value of COL1A1. Furthermore, we investigated the correlation between COL1A1, CD276 and B7-H4 in TCGA lung tumor cohort, which indicated there was a close positive correlation between COL1A1 and CD276 (*R* = 0.39, *P* < 2.2*e* − 16) (Figs. 6B–6C). To validate the positive correlation, TIMER and GEPIA were also used to evaluate the correlation between COL1A1 and CD276 in LUAD and LUSC, with R value of 0.561, 0.361, 0.48 and 0.35, respectively (*P* < 0.001) (Figs. 6D–6H).

### COL1A1 is positively correlated with CD276

**Figure 7:**
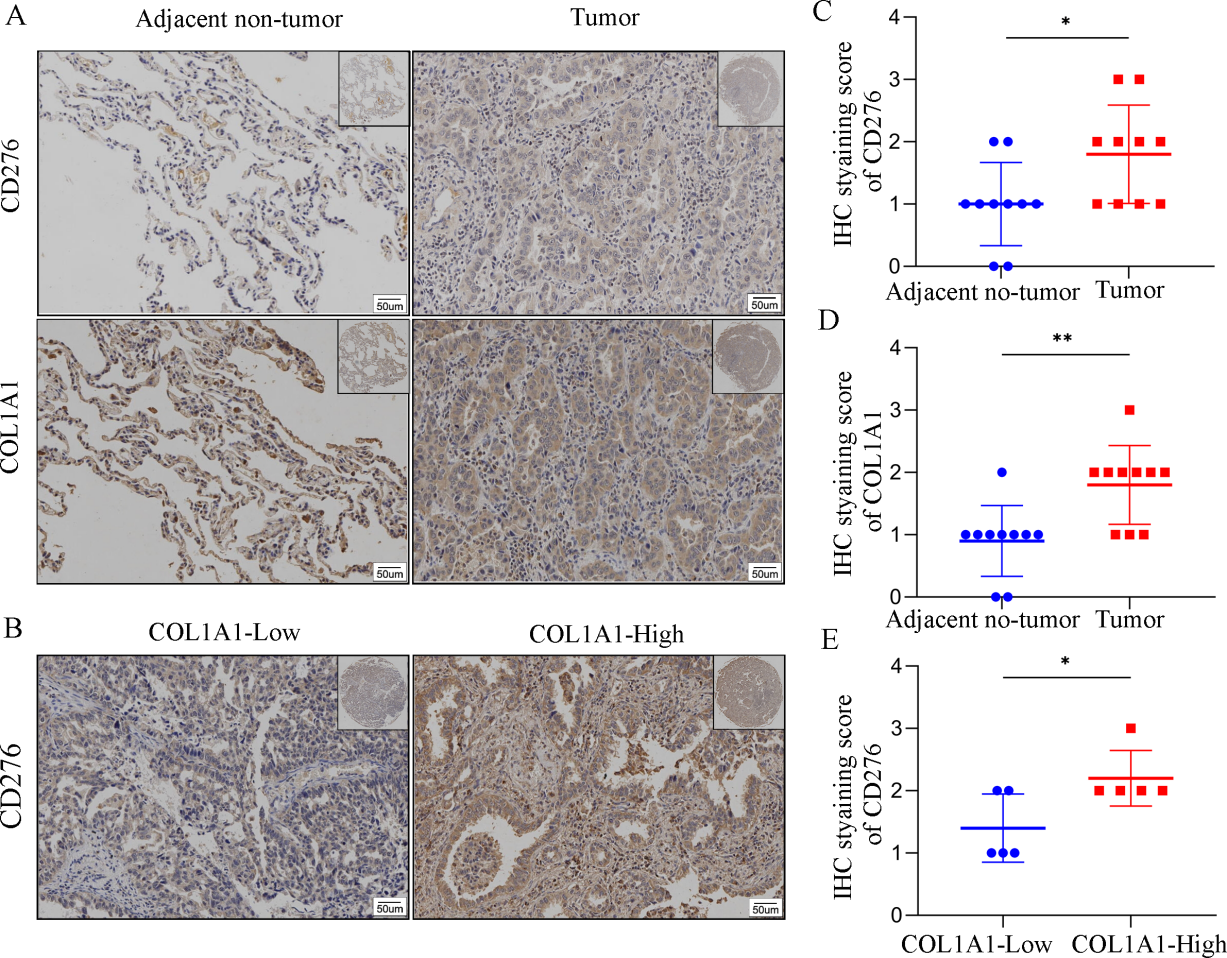
The correlation between COL1A1 and CD276 in LC. (A)Tumors and adjacent no-tumor tissues from a cohort of patients with LC were stained for COL1A1 expression at the protein level. (B) Tumors and adjacent no-tumor tissues from a cohort of patients with LC were stained for CD276 expression at the protein level. (C) CD276 showed high expression in tumor tissues with high COL1A1 expression. IHC score of COL1A1(D) and CD276 (E) in adjacent no-tumor tissues and tumor tissues from patients with LC. (F) IHC score of CD276 in tumor tissues form COL1A1-high and low patient groups, ^∗^*p* < 0.05, ^∗∗^*P* < 0.01.

In order to assess COL1A1 correlation with CD276, we analyzed COL1A1 and CD276 expression in tumor sites and the adjacent non-tumor samples (Fig. 7A). We found that COL1A1 and CD276 showed significantly higher expression in tumor sites than in the adjacent non-tumor samples (Figs. 7C–7D). Furthermore, we divided the tumor sites into two groups according to COL1A1 expression and found that CD276 levels were positively correlated with the level of COL1A1(Fig. 7B, Fig. 7E).

## Discussion

COL1A1 is involved in encoding type I collagen, which is a member of collagens family that regulate intercellular adhesion and differentiation and strengthen many tissues in the body (Marini et al., 2017). In addition, collagen is the major structural protein of extracellular matrix (ECM), which is an important part of the tumor microenvironment, and plays a vital role in tumor formation, metastasis and development (Lu, Weaver & Werb, 2012). Previous studies have shown that COL1A1 can participate in glutamine-mediated interaction between pancreatic cancer and stellate cells (Chakravarthy et al., 2018), and silencing COL1A1(siCOLIA1) inhibited the orthotopic growth of colorectal tumor in mice (Wu et al., 2020). Besides, cell experiment indicates, siCOLIA1 suppressed hepatocellular carcinoma (HCC) cells clonogenicity, motility, invasiveness and tumorsphere formation (Ma, Chang & Bamodu, 2019). These findings suggest COL1A1 plays an important role in the formation and metastasis of various tumors. However, the distinct role of COL1A1 in malignant, pre-malignant and normal tissues and its clinical significance in LC are unclear. In this study, we found that 340 genes were significantly differentially expressed in normal tissues and lung cancer tissues through genetic screening. After PPI network and Lasso regression analysis, seven genes (COL1A1, SPP1, TIMP1, PECAM1, CAV1, PPARG and CDN1) were identified as components of the risk signature to divide LC into low and high-risk groups. Kaplan–Meier Plotter also showed a high COL1A1 expression correlated with high hazard ratio (HR) for OS and PFS of lung tumor. In addition, TCGA, GSE31210 and GSE3141 datasets were used to validate the prognostic value of COL1A1, suggesting that COL1A1 played a significant part in the prognostic of lung tumor patients.

LC, the leading cause of cancer death in the worldwide, is the main part of cancer death in both men and women and accounts for a whopping 18% of all cancer deaths (Sung, Ferlay & Siegel, 2021). Emerging studies have shown Immunotherapy is a successful treatment for lung cancer. However, most adverse reactions are caused by Immunotherapy, which can be relieve by symptomatic treatment, application of glucocorticoids and related monoclonal antibodies. In addition, like other anti-tumor drugs, immunotherapy will inevitably produce drug resistance (O’Donnell, Teng & Smyth, 2019; Sharma et al., 2017). Therefore, finding new immune-related genes is crucial. The co-suppression pathway in the B7-CD28 family can provide key inhibitory signals, thereby regulating immune homeostasis and defense capabilities and protecting tissue integrity (Janakiram et al., 2017). Previous studies have shown that the immune microenvironment plays a vital role in tumorigenesis and development. Therefore, immune-related biomarkers have also attracted much attention in risk stratification and prognosis prediction (Pagni et al., 2019).

More importantly, COL1A1 expression has significant correlations with diverse immune infiltration levels and many immune marker sets in LUAD and LUSC. In addition, the study shows that COL1A1 expression is closely related with tumor purity and linked to CD4+ T cells, Macrophage, Neutrophil and Dendritic cell in LUAD and LUSC. Furthermore, the correlation between COL1A1 and the B7-CD28 ligand–receptor family was analyzed based on TCGA database, indicating there was a close positive correlation between COL1A1 and CD276, which was consistent with the prognostic value of COL1A1. Previous study shown that COL1A1 was correlated positively with the tumor infiltration levels of CD4+ T cells and macrophages in Bladder Cancer (Li et al., 2020). CD276, expressed in multiple tumor lines, tumor-infiltrating dendritic cells, and macrophages, can inhibit T-cell activation and autoimmunity (Son, Kwon & Cho, 2019). Therefore, the interaction between COL1A1 and CD276 could be a potential mechanism for the correlation among COL1A1 expression, immune infiltration and poor prognosis in LC.

## Conclusions

In summary, increased COL1A1 expression correlated with poor prognosis and changed immune infiltration levels in LC, indicating COL1A1 is a potential immunity-related biomarker. This study was based on statistical analysis of bioinformatics methods, and the conclusions obtained had experimental support and multiple database verification. Thus, our study provides insights in understanding the potential role of COLA1 in tumor immunology and its use as a cancer biomarker.

##  Supplemental Information

10.7717/peerj.11145/supp-1 Supplemental Information 1 The information of 340 common DEGs

10.7717/peerj.11145/supp-2 Supplemental Information 2 Construction of hub genes prognostic classifier (A, B) Determination of the number of factors by the LASSO analysis. Each curve represents the change track of the coefficient of each independent variable. (C) The ROC applied to evaluate the accuracy and discrimination of modeling. (D) the distribution of risk score, survival status and gene expression panel. The black dotted line is the optimal cut-off value for dividing patients into low-risk and high-risk groups.

10.7717/peerj.11145/supp-3 Supplemental Information 3 The immune infiltration analysis in LUAD (A) and LUSC (B)

10.7717/peerj.11145/supp-4 Supplemental Information 4 The information of 340 common DEGs

## Additional Information and Declarations

### Data Availability

The following information was supplied regarding data availability: Data is available at NCBI GEO: GSE3141, GSE31210, GSE32867, GSE43458 and GSE116959. TCGA Lung Cancer dataset is available at the Cancer Genomics Browser of the University of California Santa Cruz: https://xenabrowser.net/datapages/?cohort=TCGA%20Lung%20Cancer%20(LUNG)removeHub=https%3A%2F%2Fxena.treehouse.gi.ucsc.edu%3A443.

## References

[ref-1] Altan M, Pelekanou V, Schalper KA, Toki M, Gaule P, Syrigos K, Herbst RS, Rimm DL (2017). B7-H3 expression in NSCLC and its association with B7-H4, PD-L1 and tumor-infiltrating lymphocytes. Clinical Cancer Research.

[ref-2] Bindea G, Mlecnik B, Hackl H, Charoentong P, Tosolini M, Kirilovsky A, Fridman WH, Pagès F, Trajanoski Z, Galon J (2009). ClueGO: a Cytoscape plug-in to decipher functionally grouped gene ontology and pathway annotation networks. Bioinformatics.

[ref-3] Bray F, Ferlay J, Soerjomataram I, Siegel RL, Torre LA, Jemal A (2018). Global cancer statistics 2018: GLOBOCAN estimates of incidence and mortality worldwide for 36 cancers in 185 countries. CA: A Cancer Journal for Clinicians.

[ref-4] Chakravarthy D, Muñoz AR, Su A, Hwang RF, Keppler BR, Chan DE, Halff G, Ghosh R, Kumar AP (2018). Palmatine suppresses glutamine-mediated interaction between pancreatic cancer and stellate cells through simultaneous inhibition of survivin and COL1A1. Cancer Letters.

[ref-5] Chin CH, Chen SH, Wu HH, Ho CW, Ko MT, Lin CY (2014). cytoHubba: identifying hub objects and sub-networks from complex interactome. BMC Systems Biology.

[ref-6] Ferlay J, Colombet M, Soerjomataram I, Mathers C, Parkin DM (2019). Estimating the global cancer incidence and mortality in 2018: GLOBOCAN sources and methods. International Journal of Cancer.

[ref-7] Fesnak AD, June CH, Levine BL (2016). Engineered T cells: the promise and challenges of cancer immunotherapy. Nature Reviews Cancer.

[ref-8] Hellmann MD, Ciuleanu TE, Pluzanski A, Lee JS, Otterson GA, Audigier-Valette C, Minenza E, Linardou H, Burgers S, Salman P, Borghaei H, Ramalingam SS, Brahmer J, Reck M, O’Byrne KJ, Geese WJ, Green G, Chang H, Szustakowski J, Bhagavatheeswaran P, Healey D, Fu Y, Nathan F, Paz-Ares L (2018). Nivolumab plus ipilimumab in lung cancer with a high tumor mutational burden. New England Journal of Medicine.

[ref-9] Janakiram M, Shah UA, Liu W, Zhao A, Schoenberg MP, Zang X (2017). The third group of the B7-CD28 immune checkpoint family: HHLA2, TMIGD2, B7x, and B7-H3. Immunological Reviews.

[ref-10] Kabbout M, Garcia MM, Fujimoto J, Liu DD, Woods D, Chow CW, Mendoza G, Momin AA, James BP, Solis L, Behrens C, Lee JJ, Wistuba II, Kadara H (2013). ETS2 mediated tumor suppressive function and MET oncogene inhibition in human non-small cell lung cancer. Clinical Cancer Research.

[ref-11] Li F, Teng H, Liu M, Liu B, Zhang D, Xu Z, Wang Y, Zhou H (2020). Prognostic value of immune-related genes in the tumor microenvironment of bladder cancer. Frontiers in Oncology.

[ref-12] Li T, Fan J, Wang B, Traugh N, Chen Q, Liu JS, Li B, Liu XS (2017). TIMER: a web server for comprehensive analysis of tumor-infiltrating immune cells. Cancer Research.

[ref-13] Liu J, Shen JX, Wu HT, Li XL, Wen XF, Du CW, Zhang GJ (2018). Collagen 1A1 (COL1A1) promotes metastasis of breast cancer and is a potential therapeutic target. Discovery Medicine.

[ref-14] Lu P, Weaver VM, Werb Z (2012). The extracellular matrix: a dynamic niche in cancer progression. Journal of Cell Biology.

[ref-15] Ma HP, Chang HL, Bamodu OA (2019). Collagen 1A1 (COL1A1) is a reliable biomarker and putative therapeutic target for hepatocellular carcinogenesis and metastasis. Cancers.

[ref-16] Marini JC, Forlino A, Bächinger HP, Bishop NJ, Byers PH, Paepe A, Fassier F, Fratzl-Zelman N, Kozloff KM, Krakow D, Montpetit K, Semler O (2017). Osteogenesis imperfecta. Nature Reviews Disease Primers.

[ref-17] MorenoLeon L, Gautier M, Allan R, Ilié M (2019). The nuclear hypoxia-regulated NLUCAT1 long non-coding RNA contributes to an aggressive phenotype in lung adenocarcinoma through regulation of oxidative stress. Oncogene.

[ref-18] O’Donnell JS, Teng MWL, Smyth MJ (2019). Cancer immunoediting and resistance to T cell-based immunotherapy. Nature Reviews Clinical Oncology.

[ref-19] Oleksiewicz U, Liloglou T, Tasopoulou KM, Daskoulidou N, Gosney JR, Field JK, Xinarianos G (2017). COL1A1, PRPF40A, and UCP2 correlate with hypoxia markers in non-small cell lung cancer. Journal of Cancer Research and Clinical Oncology.

[ref-20] Pagni F, Guerini-Rocco E, Schultheis AM, Grazia G (2019). Targeting immune-related biological processes in solid tumors: we do need biomarkers, 20.

[ref-21] Rocco G, Pennazza G, Santonico M, Longo F, Rocco R, Crucitti P, AntonelliIncalzi R (2018). Breathprinting and early diagnosis of lung cancer. Journal of Thoracic Oncology.

[ref-22] Schildberg FA, Klein SR, Freeman GJ, Sharpe AH (2016). Coinhibitory pathways in the B7-CD28 ligand-receptor family. Immunity.

[ref-23] Selamat SA, Chung BS, Girard L, Zhang W, Zhang Y, Campan M, Siegmund KD, Koss MN, Hagen JA, Lam WL, Lam S, Gazdar AF, Laird-Offringa IA (2012). Genome-scale analysis of DNA methylation in lung adenocarcinoma and integration with mRNA expression. Genome Research.

[ref-24] Sharma P, Hu-Lieskovan S, Wargo JA, Ribas A (2017). Primary, adaptive, and acquired resistance to cancer immunotherapy. Cell.

[ref-25] Sharpe AH, Freeman GJ (2002). The B7-CD28 superfamily. Nature Reviews Immunology.

[ref-26] Smoot ME, Ono K, Ruscheinski J, Wang PL, Ideker T (2011). Cytoscape 2.8: new features for data integration and network visualization. Bioinformatics.

[ref-27] Son Y, Kwon SM, Cho JY (2019). CD276 (B7-H3) maintains proliferation and regulates differentiation in angiogenic function in late endothelial progenitor cells. Stem Cells.

[ref-28] Sung H, Ferlay J, Siegel RL (2021). Global cancer statistics 2020: GLOBOCAN estimates of incidence and mortality worldwide for 36 cancers in 185 countries. CA: A Cancer Journal for Clinicians.

[ref-29] Szklarczyk D, Gable AL, Lyon D, Junge A, Wyder S, Huerta-Cepas J, Simonovic M, Doncheva NT, Morris JH, Bork P, Jensen LJ, Mering CV (2019). STRING v11: protein-protein association networks with increased coverage, supporting functional discovery in genome-wide experimental datasets. Nucleic Acids Research.

[ref-30] Tanaka A, Sakaguchi S (2017). Regulatory T cells in cancer immunotherapy. Cell Research.

[ref-31] Tang Z, Li C, Kang B, Gao G, Li C, Zhang Z (2017). GEPIA: a web server for cancer and normal gene expression profiling and interactive analyses. Nucleic Acids Research.

[ref-32] Wong JYY, Downward GS, Hu W, Portengen L, Seow WJ, Silverman DT, Bassig BA (2019). Lung cancer risk by geologic coal deposits: a case-control study of female never-smokers from Xuanwei and Fuyuan, China. International Journal of Cancer.

[ref-33] Wu W, Yang Z, Long F, Luo L, Deng Q, Wu J, Ouyang S, Tang D (2020). COL1A1 and MZB1 as the hub genes influenced the proliferation, invasion, migration and apoptosis of rectum adenocarcinoma cells by weighted correlation network analysis. Bioorganic Chemistry.

