# Supplementary material for: COL1A1 is a prognostic biomarker and correlated with immune infiltrates in lung cancer
Source: PeerJ. 2021 Mar 30;9:e11145. doi: 10.7717/peerj.11145 (PMC8018245; doi:10.7717/peerj.11145)
Supplement: Supplemental Information 1 [file peerj-09-11145-s001.pdf]

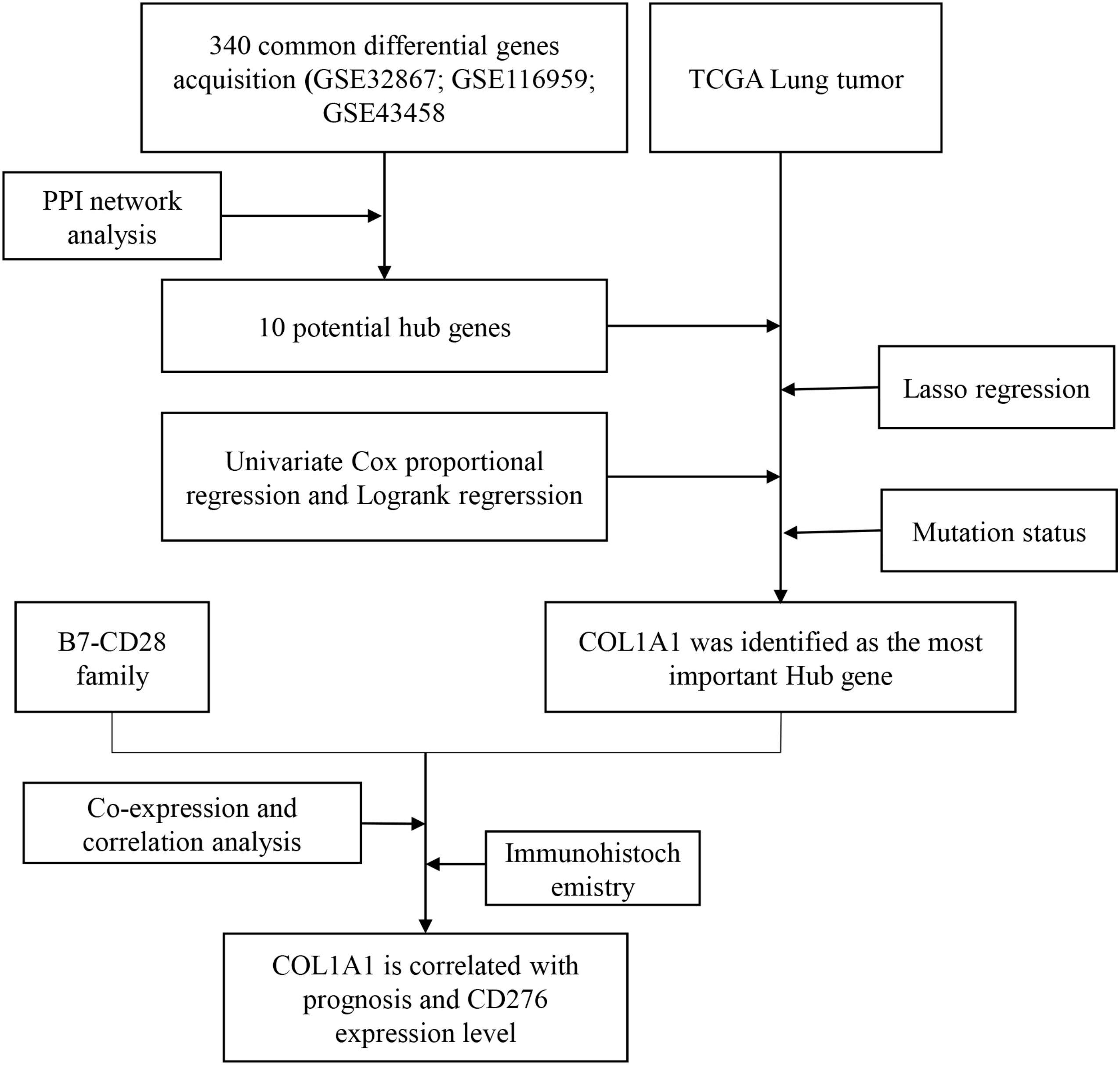
340 common differential genes acquisition (GSE32867; GSE116959; GSE43458
TCGA Lung tumor
PPI network analysis
10 potential hub genes
Lasso regression
Univariate Cox proportional regression and Logrank regrerssion
Mutation status
B7-CD28 family
COL1A1 was identified as the most important Hub gene
Co-expression and correlation analysis
Immunohistoch emistry
COL1A1 is correlated with prognosis and CD276 expression level
