# Supplementary figures and images for: COL1A1 is a prognostic biomarker and correlated with immune infiltrates in lung cancer
Source: PeerJ. 2021 Mar 30;9:e11145. doi: 10.7717/peerj.11145 (PMC8018245; doi:10.7717/peerj.11145)

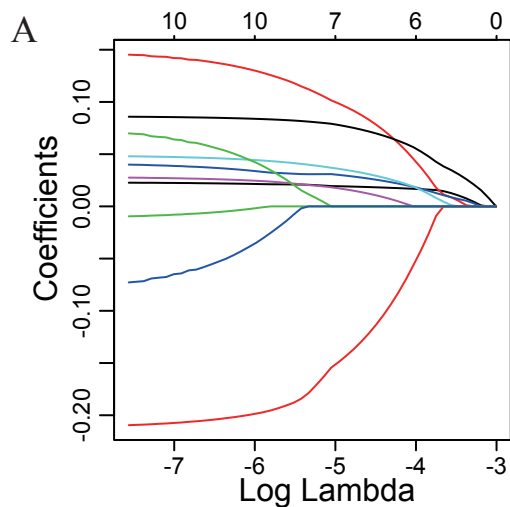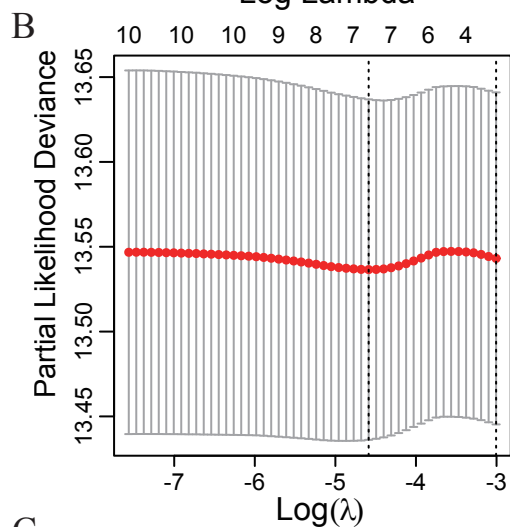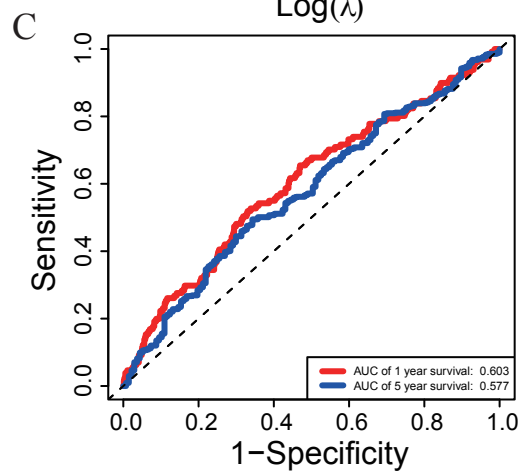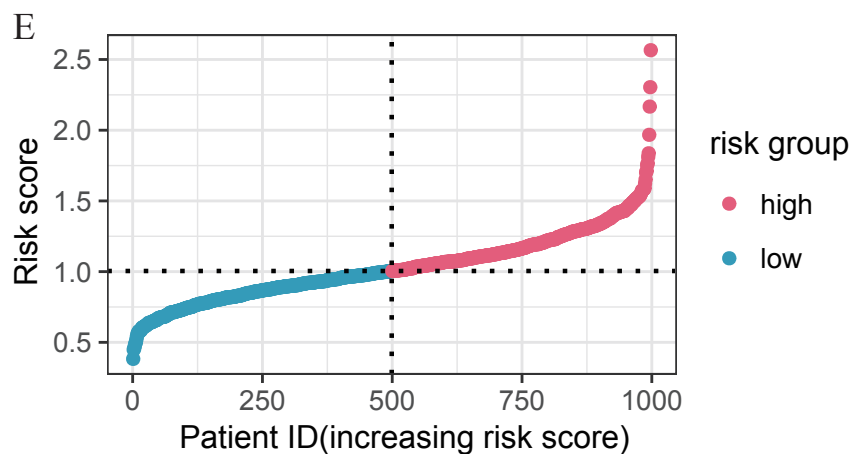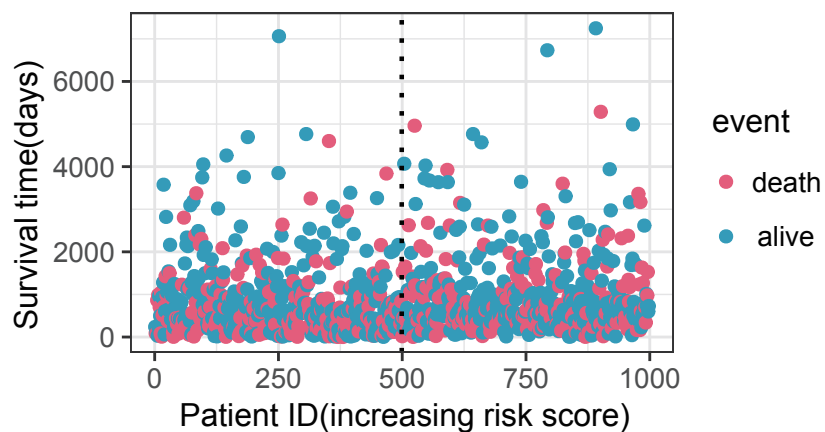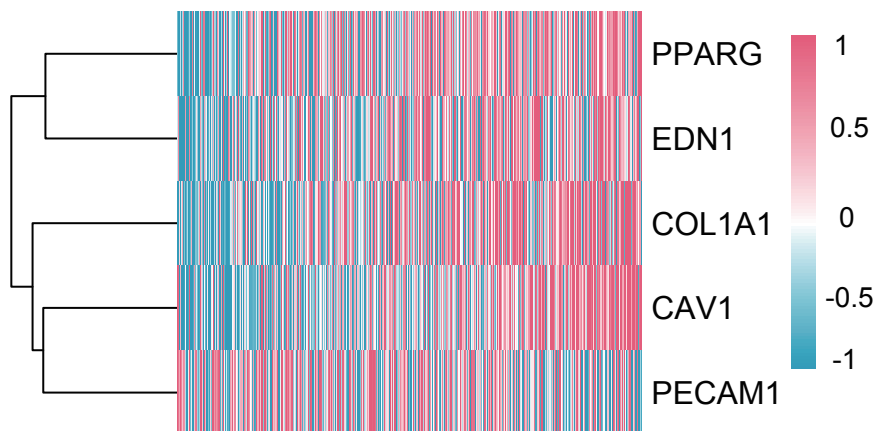

Supplement: Supplemental Information 2 — (A, B) Determination of the number of factors by the LASSO analysis. Each curve represents the change track of the coefficient of each independent variable. (C) The ROC applied to evaluate the accuracy and discrimination of modeling. (D) the distribution of risk score, survival status and gene expression panel. The black dotted line is the optimal cut-off value for dividing patients into low-risk and high-risk groups. [file peerj-09-11145-s002.pdf]

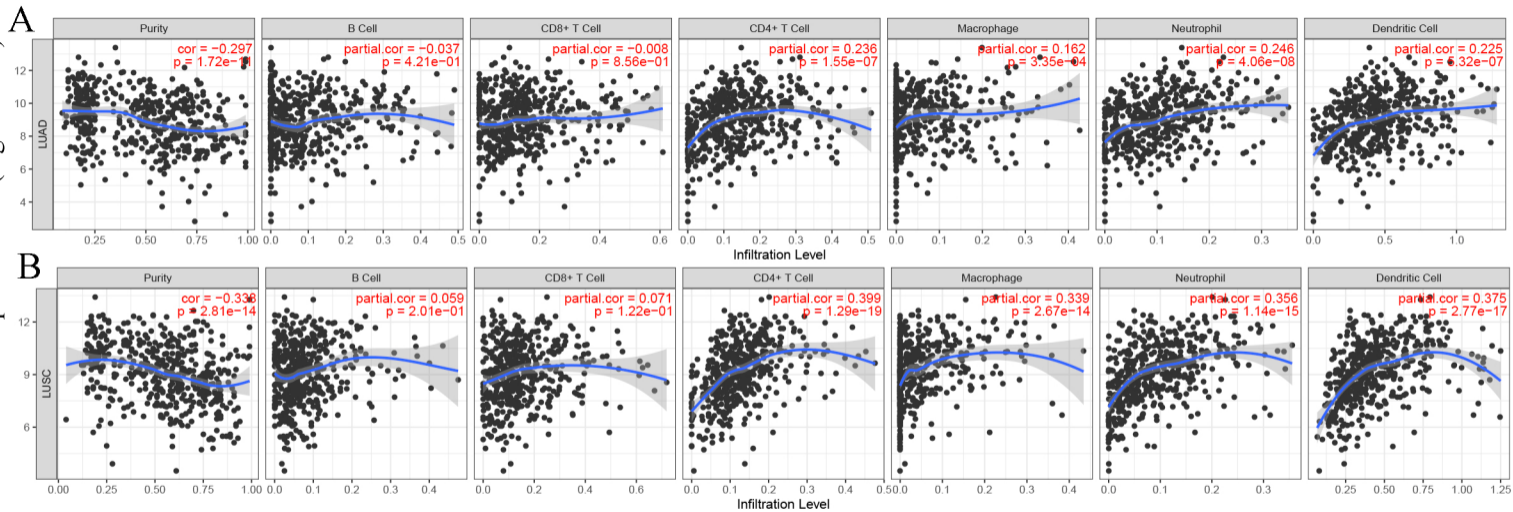

Supplement: Supplemental Information 3 [file peerj-09-11145-s003.pdf]
