# Supplementary material for: COL1A1 is a prognostic biomarker and correlated with immune infiltrates in lung cancer
Source: PeerJ. 2021 Mar 30;9:e11145. doi: 10.7717/peerj.11145 (PMC8018245; doi:10.7717/peerj.11145)
Supplement: Supplemental Information 4 [file peerj-09-11145-s004.docx]

| **Gene Symbol** | **Gene Name** | **Expression in Tumor** |
| --- | --- | --- |
| PYCR1 | pyrroline-5-carboxylate reductase 1 | Up |
| ETV4 | ETS variant 4 | Up |
| OCIAD2 | OCIA domain containing 2 | Up |
| TOP2A | topoisomerase (DNA) II alpha | Up |
| PROM2 | prominin 2 | Up |
| MMP11 | matrix metallopeptidase 11 | Up |
| SLC2A1 | solute carrier family 2 member 1 | Up |
| TIMP1 | TIMP metallopeptidase inhibitor 1 | Up |
| SPP1 | secreted phosphoprotein 1 | Up |
| STIL | SCL/TAL1 interrupting locus | Up |
| CDC20 | cell division cycle 20 | Up |
| TNFRSF21 | TNF receptor superfamily member 21 | Up |
| SFN | stratifin | Up |
| GOLM1 | golgi membrane protein 1 | Up |
| LAD1 | ladinin 1 | Up |
| PRC1 | protein regulator of cytokinesis 1 | Up |
| CRABP2 | cellular retinoic acid binding protein 2 | Up |
| COL1A1 | collagen type I alpha 1 chain | Up |
| SMPDL3B | sphingomyelin phosphodiesterase acid like 3B | Up |
| UBE2T | ubiquitin conjugating enzyme E2 T | Up |
| SLC50A1 | solute carrier family 50 member 1 | Up |
| CCNB2 | cyclin B2 | Up |
| ANKRD22 | ankyrin repeat domain 22 | Up |
| FAM83A | family with sequence similarity 83 member A | Up |
| CDH3 | cadherin 3 | Up |
| MELK | maternal embryonic leucine zipper kinase | Up |
| KIF20A | kinesin family member 20A | Up |
| ASPM | abnormal spindle microtubule assembly | Up |
| EPCAM | epithelial cell adhesion molecule | Up |
| CENPF | centromere protein F | Up |
| FUT3 | fucosyltransferase 3 (Lewis blood group) | Up |
| SPDEF | SAM pointed domain containing ETS transcription factor | Up |
| METTL7B | methyltransferase like 7B | Up |
| FUT2 | fucosyltransferase 2 | Up |
| FBXO32 | F-box protein 32 | Up |
| PSAT1 | phosphoserine aminotransferase 1 | Up |
| PLEK2 | pleckstrin 2 | Up |
| CDC45 | cell division cycle 45 | Up |
| THBS2 | thrombospondin 2 | Up |
| KIAA0101 | KIAA0101 | Up |
| MMP9 | matrix metallopeptidase 9 | Up |
| TMPRSS4 | transmembrane protease, serine 4 | Up |
| ECT2 | epithelial cell transforming 2 | Up |
| HMGB3 | high mobility group box 3 | Up |
| COL10A1 | collagen type X alpha 1 chain | Up |
| CTHRC1 | collagen triple helix repeat containing 1 | Up |
| COMP | cartilage oligomeric matrix protein | Up |
| KDELR3 | KDEL endoplasmic reticulum protein retention receptor 3 | Up |
| TPX2 | TPX2, microtubule nucleation factor | Up |
| DSP | desmoplakin | Up |
| AK4 | adenylate kinase 4 | Up |
| CDCA7 | cell division cycle associated 7 | Up |
| SLC7A5 | solute carrier family 7 member 5 | Up |
| SPINK1 | serine peptidase inhibitor, Kazal type 1 | Up |
| CEACAM5 | carcinoembryonic antigen related cell adhesion molecule 5 | Up |
| TYMS | thymidylate synthetase | Up |
| NQO1 | NAD(P)H quinone dehydrogenase 1 | Up |
| COL3A1 | collagen type III alpha 1 chain | Up |
| GJB2 | gap junction protein beta 2 | Up |
| MMP12 | matrix metallopeptidase 12 | Up |
| AGR2 | anterior gradient 2, protein disulphide isomerase family member | Up |
| RPL39L | ribosomal protein L39 like | Up |
| SULF1 | sulfatase 1 | Up |
| IGF2BP3 | insulin like growth factor 2 mRNA binding protein 3 | Up |
| AIM2 | absent in melanoma 2 | Up |
| C15orf48 | chromosome 15 open reading frame 48 | Up |
| CHRNA5 | cholinergic receptor nicotinic alpha 5 subunit | Up |
| LGSN | lengsin, lens protein with glutamine synthetase domain | Up |
| CLDN10 | claudin 10 | Up |
| PCP4 | Purkinje cell protein 4 | Up |
| S100P | S100 calcium binding protein P | Up |
| TCN1 | transcobalamin 1 | Up |
| FABP4 | fatty acid binding protein 4 | Down |
| ADH1B | alcohol dehydrogenase 1B (class I), beta polypeptide | Down |
| ITLN2 | intelectin 2 | Down |
| FMO2 | flavin containing monooxygenase 2 | Down |
| AGER | advanced glycosylation end-product specific receptor | Down |
| FAM107A | family with sequence similarity 107 member A | Down |
| CLEC3B | C-type lectin domain family 3 member B | Down |
| CLEC1A | C-type lectin domain family 1 member A | Down |
| TCF21 | transcription factor 21 | Down |
| CAV1 | caveolin 1 | Down |
| STX11 | syntaxin 11 | Down |
| RHOJ | ras homolog family member J | Down |
| JAM2 | junctional adhesion molecule 2 | Down |
| LDB2 | LIM domain binding 2 | Down |
| EDNRB | endothelin receptor type B | Down |
| TEK | TEK receptor tyrosine kinase | Down |
| ABCA8 | ATP binding cassette subfamily A member 8 | Down |
| PECAM1 | platelet and endothelial cell adhesion molecule 1 | Down |
| LYVE1 | lymphatic vessel endothelial hyaluronan receptor 1 | Down |
| S1PR1 | sphingosine-1-phosphate receptor 1 | Down |
| SPTBN1 | spectrin beta, non-erythrocytic 1 | Down |
| CYYR1 | cysteine and tyrosine rich 1 | Down |
| CA4 | carbonic anhydrase 4 | Down |
| SLIT3 | slit guidance ligand 3 | Down |
| MMRN1 | multimerin 1 | Down |
| GHR | growth hormone receptor | Down |
| MME | membrane metallo-endopeptidase | Down |
| FHL1 | four and a half LIM domains 1 | Down |
| MFAP4 | microfibrillar associated protein 4 | Down |
| SPOCK2 | sparc/osteonectin, cwcv and kazal-like domains proteoglycan (testican) 2 | Down |
| ADH1A | alcohol dehydrogenase 1A (class I), alpha polypeptide | Down |
| HIGD1B | HIG1 hypoxia inducible domain family member 1B | Down |
| CD36 | CD36 molecule | Down |
| ADARB1 | adenosine deaminase, RNA specific B1 | Down |
| HBB | hemoglobin subunit beta | Down |
| GSTM5 | glutathione S-transferase mu 5 | Down |
| PTPRB | protein tyrosine phosphatase, receptor type B | Down |
| OLFML1 | olfactomedin like 1 | Down |
| CRYAB | crystallin alpha B | Down |
| LAMP3 | lysosomal associated membrane protein 3 | Down |
| GLIPR2 | GLI pathogenesis related 2 | Down |
| CD93 | CD93 molecule | Down |
| CPA3 | carboxypeptidase A3 | Down |
| COL6A6 | collagen type VI alpha 6 chain | Down |
| TGFBR3 | transforming growth factor beta receptor 3 | Down |
| SRPX | sushi repeat containing protein, X-linked | Down |
| MAMDC2 | MAM domain containing 2 | Down |
| AFF3 | AF4/FMR2 family member 3 | Down |
| SPARCL1 | SPARC like 1 | Down |
| HEG1 | heart development protein with EGF like domains 1 | Down |
| VWF | von Willebrand factor | Down |
| ABI3BP | ABI family member 3 binding protein | Down |
| FBLN5 | fibulin 5 | Down |
| SDPR | serum deprivation response | Down |
| CDO1 | cysteine dioxygenase type 1 | Down |
| EMCN | endomucin | Down |
| EPAS1 | endothelial PAS domain protein 1 | Down |
| ACVRL1 | activin A receptor like type 1 | Down |
| PGM5 | phosphoglucomutase 5 | Down |
| VSIG4 | V-set and immunoglobulin domain containing 4 | Down |
| FEZ1 | fasciculation and elongation protein zeta 1 | Down |
| FAM150B | family with sequence similarity 150 member B | Down |
| CRTAC1 | cartilage acidic protein 1 | Down |
| STARD13 | StAR related lipid transfer domain containing 13 | Down |
| ITM2A | integral membrane protein 2A | Down |
| PTPRM | protein tyrosine phosphatase, receptor type M | Down |
| MS4A2 | membrane spanning 4-domains A2 | Down |
| GPC3 | glypican 3 | Down |
| FGD5 | FYVE, RhoGEF and PH domain containing 5 | Down |
| TNNC1 | troponin C1, slow skeletal and cardiac type | Down |
| CLEC14A | C-type lectin domain family 14 member A | Down |
| AQP4 | aquaporin 4 | Down |
| PCOLCE2 | procollagen C-endopeptidase enhancer 2 | Down |
| GRK5 | G protein-coupled receptor kinase 5 | Down |
| ANGPT1 | angiopoietin 1 | Down |
| BCHE | butyrylcholinesterase | Down |
| GPM6B | glycoprotein M6B | Down |
| SLC19A3 | solute carrier family 19 member 3 | Down |
| PEBP4 | phosphatidylethanolamine binding protein 4 | Down |
| LGI3 | leucine rich repeat LGI family member 3 | Down |
| TMEM100 | transmembrane protein 100 | Down |
| ACADL | acyl-CoA dehydrogenase, long chain | Down |
| GKN2 | gastrokine 2 | Down |
| KLF4 | Kruppel like factor 4 | Down |
| DCN | decorin | Down |
| SEMA6A | semaphorin 6A | Down |
| FAM189A2 | family with sequence similarity 189 member A2 | Down |
| NDRG2 | NDRG family member 2 | Down |
| GIMAP4 | GTPase, IMAP family member 4 | Down |
| EML1 | echinoderm microtubule associated protein like 1 | Down |
| FZD4 | frizzled class receptor 4 | Down |
| SEPP1 | selenoprotein P, plasma, 1 | Down |
| CALCRL | calcitonin receptor like receptor | Down |
| PID1 | phosphotyrosine interaction domain containing 1 | Down |
| CAT | catalase | Down |
| HOXA5 | homeobox A5 | Down |
| TACC1 | transforming acidic coiled-coil containing protein 1 | Down |
| RAMP3 | receptor (G protein-coupled) activity modifying protein 3 | Down |
| AOC3 | amine oxidase, copper containing 3 | Down |
| C10orf67 | chromosome 10 open reading frame 67 | Down |
| CLDN18 | claudin 18 | Down |
| TIMP3 | TIMP metallopeptidase inhibitor 3 | Down |
| LPL | lipoprotein lipase | Down |
| FERMT2 | fermitin family member 2 | Down |
| IL33 | interleukin 33 | Down |
| CD34 | CD34 molecule | Down |
| SOSTDC1 | sclerostin domain containing 1 | Down |
| CGNL1 | cingulin like 1 | Down |
| GIMAP8 | GTPase, IMAP family member 8 | Down |
| MARCO | macrophage receptor with collagenous structure | Down |
| AQP9 | aquaporin 9 | Down |
| HBEGF | heparin binding EGF like growth factor | Down |
| PPP1R15A | protein phosphatase 1 regulatory subunit 15A | Down |
| MSRB3 | methionine sulfoxide reductase B3 | Down |
| DUOX1 | dual oxidase 1 | Down |
| RECK | reversion inducing cysteine rich protein with kazal motifs | Down |
| C2orf40 | chromosome 2 open reading frame 40 | Down |
| RAMP2 | receptor activity modifying protein 2 | Down |
| STXBP6 | syntaxin binding protein 6 | Down |
| TGFBR2 | transforming growth factor beta receptor 2 | Down |
| SESN1 | sestrin 1 | Down |
| RTKN2 | rhotekin 2 | Down |
| MSR1 | macrophage scavenger receptor 1 | Down |
| HHIP | hedgehog interacting protein | Down |
| FCN3 | ficolin 3 | Down |
| LMCD1 | LIM and cysteine rich domains 1 | Down |
| RRAS | related RAS viral (r-ras) oncogene homolog | Down |
| PDE5A | phosphodiesterase 5A | Down |
| VGLL3 | vestigial like family member 3 | Down |
| ARHGAP44 | Rho GTPase activating protein 44 | Down |
| TMEM47 | transmembrane protein 47 | Down |
| PDZD2 | PDZ domain containing 2 | Down |
| WASF3 | WAS protein family member 3 | Down |
| CPB2 | carboxypeptidase B2 | Down |
| TMEM204 | transmembrane protein 204 | Down |
| CACNA2D2 | calcium voltage-gated channel auxiliary subunit alpha2delta 2 | Down |
| PDK4 | pyruvate dehydrogenase kinase 4 | Down |
| KIAA1324L | KIAA1324 like | Down |
| LRRC32 | leucine rich repeat containing 32 | Down |
| LHFP | lipoma HMGIC fusion partner | Down |
| GNG11 | G protein subunit gamma 11 | Down |
| SASH1 | SAM and SH3 domain containing 1 | Down |
| OLR1 | oxidized low density lipoprotein receptor 1 | Down |
| S100A8 | S100 calcium binding protein A8 | Down |
| DPT | dermatopontin | Down |
| MYH10 | myosin, heavy chain 10, non-muscle | Down |
| PTRF | polymerase I and transcript release factor | Down |
| C7 | complement component 7 | Down |
| PLSCR4 | phospholipid scramblase 4 | Down |
| EFEMP1 | EGF containing fibulin like extracellular matrix protein 1 | Down |
| PPARG | peroxisome proliferator activated receptor gamma | Down |
| ESAM | endothelial cell adhesion molecule | Down |
| CDH13 | cadherin 13 | Down |
| SEMA5A | semaphorin 5A | Down |
| MAOB | monoamine oxidase B | Down |
| CBX7 | chromobox 7 | Down |
| ADCY4 | adenylate cyclase 4 | Down |
| SOCS2 | suppressor of cytokine signaling 2 | Down |
| SLC39A8 | solute carrier family 39 member 8 | Down |
| NTNG1 | netrin G1 | Down |
| FPR2 | formyl peptide receptor 2 | Down |
| CTNNAL1 | catenin alpha like 1 | Down |
| LRRN3 | leucine rich repeat neuronal 3 | Down |
| TSPAN7 | tetraspanin 7 | Down |
| AOX1 | aldehyde oxidase 1 | Down |
| RNF144B | ring finger protein 144B | Down |
| TBX3 | T-box 3 | Down |
| SLIT2 | slit guidance ligand 2 | Down |
| MYADM | myeloid associated differentiation marker | Down |
| SMAD6 | SMAD family member 6 | Down |
| GLDN | gliomedin | Down |
| DACH1 | dachshund family transcription factor 1 | Down |
| DUSP1 | dual specificity phosphatase 1 | Down |
| FGFBP2 | fibroblast growth factor binding protein 2 | Down |
| CYBRD1 | cytochrome b reductase 1 | Down |
| FMO3 | flavin containing monooxygenase 3 | Down |
| CES1 | carboxylesterase 1 | Down |
| CLIC5 | chloride intracellular channel 5 | Down |
| IL7R | interleukin 7 receptor | Down |
| NOSTRIN | nitric oxide synthase trafficking | Down |
| B3GALNT1 | beta-1,3-N-acetylgalactosaminyltransferase 1 (globoside blood group) | Down |
| LIMCH1 | LIM and calponin homology domains 1 | Down |
| ANKRD29 | ankyrin repeat domain 29 | Down |
| DPYSL2 | dihydropyrimidinase like 2 | Down |
| VIPR1 | vasoactive intestinal peptide receptor 1 | Down |
| NPNT | nephronectin | Down |
| LMO7 | LIM domain 7 | Down |
| ANXA3 | annexin A3 | Down |
| ICAM2 | intercellular adhesion molecule 2 | Down |
| CYP4B1 | cytochrome P450 family 4 subfamily B member 1 | Down |
| DES | desmin | Down |
| STARD8 | StAR related lipid transfer domain containing 8 | Down |
| HSD17B6 | hydroxysteroid 17-beta dehydrogenase 6 | Down |
| PROS1 | protein S (alpha) | Down |
| SFTPC | surfactant protein C | Down |
| ABCA3 | ATP binding cassette subfamily A member 3 | Down |
| PLA2G1B | phospholipase A2 group IB | Down |
| WIF1 | WNT inhibitory factor 1 | Down |
| PRICKLE2 | prickle planar cell polarity protein 2 | Down |
| FRAS1 | Fraser extracellular matrix complex subunit 1 | Down |
| SLC6A4 | solute carrier family 6 member 4 | Down |
| S100A3 | S100 calcium binding protein A3 | Down |
| CA2 | carbonic anhydrase 2 | Down |
| AHNAK | AHNAK nucleoprotein | Down |
| SGCE | sarcoglycan epsilon | Down |
| GABARAPL1 | GABA type A receptor associated protein like 1 | Down |
| TPPP3 | tubulin polymerization promoting protein family member 3 | Down |
| ACTG2 | actin, gamma 2, smooth muscle, enteric | Down |
| NEDD9 | neural precursor cell expressed, developmentally down-regulated 9 | Down |
| PGC | progastricsin | Down |
| FAM167A | family with sequence similarity 167 member A | Down |
| NDRG4 | NDRG family member 4 | Down |
| WFS1 | wolframin ER transmembrane glycoprotein | Down |
| CHI3L2 | chitinase 3 like 2 | Down |
| PDLIM3 | PDZ and LIM domain 3 | Down |
| GIMAP7 | GTPase, IMAP family member 7 | Down |
| ETS2 | ETS proto-oncogene 2, transcription factor | Down |
| MYL9 | myosin light chain 9 | Down |
| SLPI | secretory leukocyte peptidase inhibitor | Down |
| PTGIS | prostaglandin I2 (prostacyclin) synthase | Down |
| LRRK2 | leucine rich repeat kinase 2 | Down |
| NTN4 | netrin 4 | Down |
| KLF6 | Kruppel like factor 6 | Down |
| C8B | complement component 8, beta polypeptide | Down |
| RERG | RAS like estrogen regulated growth inhibitor | Down |
| METTL7A | methyltransferase like 7A | Down |
| SLCO2A1 | solute carrier organic anion transporter family member 2A1 | Down |
| MYH11 | myosin heavy chain 11 | Down |
| MS4A15 | membrane spanning 4-domains A15 | Down |
| C1orf198 | chromosome 1 open reading frame 198 | Down |
| SFTPD | surfactant protein D | Down |
| PAPSS2 | 3'-phosphoadenosine 5'-phosphosulfate synthase 2 | Down |
| ID1 | inhibitor of DNA binding 1, HLH protein | Down |
| ANKRD1 | ankyrin repeat domain 1 | Down |
| GBP4 | guanylate binding protein 4 | Down |
| SUSD2 | sushi domain containing 2 | Down |
| PLLP | plasmolipin | Down |
| A2M | alpha-2-macroglobulin | Down |
| RAB11FIP1 | RAB11 family interacting protein 1 | Down |
| HEY1 | hes related family bHLH transcription factor with YRPW motif 1 | Down |
| ITLN1 | intelectin 1 | Down |
| PLAC8 | placenta specific 8 | Down |
| SCGB1A1 | secretoglobin family 1A member 1 | Down |
| FLRT3 | fibronectin leucine rich transmembrane protein 3 | Down |
| LDLR | low density lipoprotein receptor | Down |
| TSPAN18 | tetraspanin 18 | Down |
| CX3CR1 | C-X3-C motif chemokine receptor 1 | Down |
| PHACTR2 | phosphatase and actin regulator 2 | Down |
| ROPN1L | rhophilin associated tail protein 1 like | Down |
| C1orf87 | chromosome 1 open reading frame 87 | Down |
| NDNF | neuron derived neurotrophic factor | Down |
| FOS | Fos proto-oncogene, AP-1 transcription factor subunit | Down |
| IL1RL1 | interleukin 1 receptor like 1 | Down |
| C20orf85 | chromosome 20 open reading frame 85 | Down |
| SELENBP1 | selenium binding protein 1 | Down |
| ZNF385B | zinc finger protein 385B | Down |
| SCGB3A2 | secretoglobin family 3A member 2 | Down |
| ZBTB16 | zinc finger and BTB domain containing 16 | Down |
| LAMA3 | laminin subunit alpha 3 | Down |
| NEDD4L | neural precursor cell expressed, developmentally down-regulated 4-like, E3 ubiquitin protein ligase | Down |
| MAOA | monoamine oxidase A | Down |
| C4BPA | complement component 4 binding protein alpha | Down |
| GJA1 | gap junction protein alpha 1 | Down |
| EDN1 | endothelin 1 | Down |
| CDHR3 | cadherin related family member 3 | Down |
| HPGD | hydroxyprostaglandin dehydrogenase 15-(NAD) | Down |
| LRRFIP1 | LRR binding FLII interacting protein 1 | Down |
| CCBE1 | collagen and calcium binding EGF domains 1 | Down |
